# Measuring resilience prospectively as the speed of affect recovery in daily life: a complex systems perspective on mental health

**DOI:** 10.1186/s12916-020-1500-9

**Published:** 2020-02-18

**Authors:** Anna Kuranova, Sanne H. Booij, Claudia Menne-Lothmann, Jeroen Decoster, Ruud van Winkel, Philippe Delespaul, Marc De Hert, Catherine Derom, Evert Thiery, Bart P. F. Rutten, Nele Jacobs, Jim van Os, Johanna T. W. Wigman, Marieke Wichers

**Affiliations:** 1 University of Groningen, University Medical Center Groningen, University Center Psychiatry (UCP) Interdisciplinary Center Psychopathology and Emotion Regulation (ICPE), Groningen, The Netherlands; 2 Department of Research and Education, Friesland Mental Health Care Services, Leeuwarden, The Netherlands; 3 Center for Integrative Psychiatry, Lentis, Groningen, The Netherlands; 4 Department of Psychiatry and Neuropsychology, School of mental health and neuroscience (MHeNS), Maastricht University, Maastricht, The Netherlands; 5 University Psychiatric Centre Sint-Kamillus, Bierbeek, Belgium; 6 KU Leuven, Department of Neurosciences, Center for Public Health Psychiatry, UPC KU Leuven, Leuven, Belgium; 7 KU Leuven, Department of Neurosciences, Center for Clinical Psychiatry, UPC KU Leuven, Leuven, Belgium; 8 Mondriaan Mental Health Care, Heerlen, The Netherlands; 9 Antwerp Health Law and Ethics Chair – AHLEC University Antwerpen, Antwerp, Belgium; 10 Centre of Human Genetics, University Hospital Leuven, KU Leuven, Leuven, Belgium; 11 Department of Obstetrics and Gynecology, Ghent University Hospital, Ghent University, Ghent, Belgium; 12 Department of Neurology, Ghent University Hospital, Ghent University, Ghent, Belgium; 13 Faculty of Psychology and Educational Sciences, Open University of the Netherlands, Heerlen, Netherlands; 14 Department of Psychosis Studies, Institute of Psychiatry, King’s Health Partners, King’s College London, London, UK; 15 Department Psychiatry, Brain Center Rudolf Magnus, Utrecht University Medical Centre, Utrecht, The Netherlands

**Keywords:** Psychological resilience, Complex systems theory, Speed of recovery, Psychopathology, Affect dynamics

## Abstract

**Introduction:**

There is growing evidence that mental disorders behave like complex dynamic systems. Complex dynamic systems theory states that a slower recovery from small perturbations indicates a loss of resilience of a system. This study is the first to test whether the speed of recovery of affect states from small daily life perturbations predicts changes in psychopathological symptoms over 1 year in a group of adolescents at increased risk for mental disorders.

**Methods:**

We used data from 157 adolescents from the TWINSSCAN study. Course of psychopathology was operationalized as the 1-year change in the Symptom Checklist-90 sum score. Two groups were defined: one with stable and one with increasing symptom levels. Time-series data on momentary daily affect and daily unpleasant events were collected 10 times a day for 6 days at baseline.

We modeled the time-lagged effect of daily unpleasant events on negative and positive affect after each unpleasant event experienced, to examine at which time point the impact of the events is no longer detectable.

**Results:**

There was a significant difference between groups in the effect of unpleasant events on negative affect 90 min after the events were reported. Stratified by group, in the Increase group, the effect of unpleasant events on both negative (*B* = 0.05, *p* < 0.01) and positive affect (*B* = − 0. 08, *p* < 0.01) was still detectable 90 min after the events, whereas in the Stable group this was not the case.

**Conclusion:**

Findings cautiously suggest that adolescents who develop more symptoms in the following year may display a slower affect recovery from daily perturbations at baseline. This supports the notion that mental health may behave according to the laws of a complex dynamic system. Future research needs to examine whether these dynamic indicators of system resilience may prove valuable for personalized risk assessment in this field.

**Electronic supplementary material:**

The online version of this article (10.1186/s12916-020-1500-9) contains supplementary material, which is available to authorized users.

## Background

Mental disorders are directly and indirectly associated with a large part of overall morbidity and mortality worldwide [1]. Once developed, many mental disorders tend to become chronic or recur [2]. Hence, prevention of these disorders is crucial.

Still, our current understanding of the development of psychopathology is limited, due to a substantial amount of different factors involved in this process (such as variations in individual differences and environmental factors) and complex, non-linear interactions between these factors. Such complexity behind psychopathological processes hampers accurate identification of people at risk. Embracing this complexity may be the way forward in understanding psychopathology and its development. A promising approach to obtain accurate risk estimations comes from the theory of complex systems. Examples of such complex systems are ecosystems, which are known to make shifts from a forest state to a swamp state, or the financial market, which can experience a sudden collapse [3, 4]. Although such changes are results of numerous mechanistic interactions, complex systems theory states that the stability of a system, i.e., how hard it is for a large change to occur, can be quantified in one characteristic: an index of resilience. This overall estimate of resilience of the system can be assessed by its capacity to recover from minor perturbations that occur. This phenomenon is called “critical slowing down” (CSD) and refers to the process whereby the system becomes increasingly slower in its capacity to recover [3, 4]. Indicators of CSD have been shown to predict (non)-critical transitions as well as gradual change in various sorts of complex systems, whether they are financial markets, oceans, climate, or brain activity [3, 5, 6]. If these principles work for psychopathology as well, we can assume that higher instability in the system (in this case, mental health), and thus lower resilience, means that it is more difficult to remain in a current healthy state and that this is related to, on average, higher levels of symptoms in the near future in this group of people.

Therefore, we expect that when speed of recovery from small perturbations is slower over time, this signals a lower stability of people’s current mental health state and, therefore, a higher likelihood of a change in the level of symptoms. Application of this approach to mental health, therefore, may help to improve personalized assessment of risk and resilience to psychopathology before new symptoms have arisen [7–10].

Supporting this line of reasoning, some previous studies examined indirect indicators of the process of critical slowing down, such as rising temporal autocorrelation and variance [3], in the micro-dynamics of affect states. These studies indeed showed that temporal autocorrelations and/or variances are increased in people with higher levels of psychopathological symptoms compared to people with lower levels of symptoms or healthy controls [11–15]. Moreover, a study by Wichers and Groot has shown on the individual level how a change in these indicators directly preceded a transition to a state with more psychopathological symptoms [16] Thus, there is initial empirical support suggesting that mental health may behave according to the laws of complex dynamic systems based on indirect measures of critical slowing down [10, 15, 17].

However, hardly any studies in psychopathology have examined the phenomenon of critical slowing down using direct measures of this process, i.e., direct measurements of the speed of recovery from minor perturbations in the system. For that, a design is needed that allows for the prospective and detailed assessment of the impact of minor perturbations in the flow of daily life on mental states. To our knowledge, only one recent study, by Vaessen and colleagues [18], examined in this way the speed of the affect recovery from daily stressors in groups with various levels of psychopathology. They found that speed of affect recovery was slower in people at early stages of psychosis compared to healthy volunteers and people with already developed psychosis. Although this study was not written explicitly from a complex systems perspective, results may support the predictions from that theory. This is because both healthy controls and people with established psychosis can be assumed to be in more stable states than those at early stages of psychosis. Therefore, as a next step, it is important to test the hypothesis that speed of recovery, as an indicator of the process of critical slowing down, indeed predicts the future development of psychopathology. The current study will therefore, for the first time, use “speed of recovery from minor perturbations to the system” as a direct dynamic indicator of the process of critical slowing down to examine whether this measure predicts future change in levels of psychopathology.

In order to examine this question, we used a sample of adolescents from the general population with relatively low levels of happy childhood experiences, representing an increased risk for psychopathology [19]. These adolescents come from the TWINSSCAN data set which includes baseline time-series data on affect states and daily unpleasant events, combined with baseline and follow-up assessments of (subclinical) psychopathology in a large sample of adolescents. Using a similar approach to measure the concept of “speed of recovery” as Vaessen and colleagues [18], we examined how quickly people recovered in terms of their experienced affect states from small negative events, reflecting minor perturbations, that happened throughout the day (e.g., spilled coffee, traffic jams).

In sum, the aim of this study is to examine whether the speed of recovery from small perturbations in daily life differs between adolescents with different future trajectories of psychopathology. We expect the speed of affect recovery from daily life unpleasant events to be slower in adolescents who will develop more psychopathological symptoms over 1 year than in adolescents who remain on similar levels of symptoms over 1 year.

## Methods

### Sample and design

Data came from the TWINSSCAN cohort [20], which comprises a subsample of 839 adolescents from the East Flanders Prospective Twin Study (EFPTS), a register of all multiple births in the Province of East Flanders, Belgium from 1964 [21, 22]. All twins from the registry between ages of 15 and 18 were invited to participate in the TWINSSCAN study. This study consisted of baseline assessments and annual follow-ups [23]. Data from questionnaires and experience sampling methodology (ESM) at baseline (T0) were used, as well as questionnaire data at 1-year follow-up (T1). Following our previous study with the same sample [24], we used the data from subjects with an above-average risk of psychopathology. Within this subsample, we identified two groups with similar baseline levels of symptoms, but different symptom trajectories over the following year (see below), resulting in the subsample of 157 individuals (see “Results” for detailed description of the selecting procedure).

All participants provided written informed consent. For those participants who were aged below 18 years, their parents/caretakers signed additional written consent. The local ethics committee (KU Leuven, Nr. B32220107766) approved the study.

### Instruments

#### Selection of individuals at increased risk

Similar to our previous study with the same subsample [24], four items of the Dutch questionnaire on adverse childhood experiences (JTV) [25] were used to assess the quality of childhood experiences, namely the items: “I had a happy childhood,” “my parents greatly loved each other,” “I got the attention that I needed,” and “my privacy was respected.” These four items were over 90% correlated with the overall score of the JTV questionnaire that was used in a previous twin sample of the EFPTS (see [26] for a description of this sample). In addition, they showed optimal variation in the studied population, as they are phrased positively. Therefore, for the current data collection, it was decided to assess only these four items, as it relieves the participants’ burden of filling out questionnaires, but retains essential information. These items were measured with a 5-point Likert scale ranging from 1 (“never”) to 5 (“very often”). These four items had good internal consistency (Cronbach alpha in our sample was 0.83 (confidence interval 0.80–0.85)). The sum score of the four items was calculated, and the individuals with the lowest range of safe and happy childhood experiences (*n* = 451) were identified with a median split. All participants in the final sample completed all four items.

#### Psychopathology trajectories

The number of general psychopathological symptoms was assessed at T0 and T1 with the Symptom Check List-90 (SCL-90) questionnaire [27] as a sum score of all 90 items. To assess the trajectory of psychopathology, the SCL-90 scores at T0 were subtracted from the SCL-90 scores at T1 for each participant. These change scores were divided into tertiles, resulting in three groups defined by a reduction (Decrease group, mean SCL-90 sum score change = − 41.48 points, *n* = 80), no change (Stable group, mean SCL-90 sum score change = − 5.14 points, *n* = 80, and an increase in symptom level (Increase group, mean SCL-90 sum score change = 25.9, *n* = 77) (see also Table 1). Furthermore, as the Decrease group reported significantly higher symptom levels at T0 than the other groups, adding this group would not help to answer the research question as we would not be able to make valid comparisons between this group and the other groups. Therefore the Decrease group was excluded from the further analysis.

**Table 1:** Sociodemographic characteristics, level of happy childhood experiences (JTV), Symptom Check List-90 scores, number of negative life events between T0 and T1, percent of twin pairs allocated to the same group, and number, mean levels, and SDs of ESM variables for the Stable and Increase groups

Measure	The Stable group			The Increase group		
Number of people	80			77		
% females	68.75%			63.64%		
Education (*n*)						
Low education	8			4		
Middle education	50			54		
High education	22			17		
No data	0			2		
Ethnicity (*n*)						
Caucasian	78			76		
Asian	1			0		
No data	1			1		
% of twin pairs allocated to the same group	9.59			14.03		
	M	SD	Range	M	SD	Range
Age at T0	17.85	3.98	14–33	16.95	3.60	15–34
JTV sum score at T0*	15.58	1.57	11–17	14.96	2.16	7–17
SCL-90 sum score at T0	127.08	26.13	92–214	130.73	33.91	90–245
SCL-90 sum score at T1*	121.94	25.89	90–212	156.65	42.18	105–305
Number of negative life events between T0 and T1	2.95	1.44	1–7	3.40	1.98	1–10
ESM measures at T0						
Number of unpleasant events	15.91	9.01	1–50	17.12	10.00	2–44
	M	SD within-person		M	SD within-person	
Level of unpleasantness of unpleasant events	0.91	0.86		0.87	0.86	
Negative affect score	1.72	0.51		1.87	0.61	
Positive affect score	4.88	0.83		4.72	0.91	

Note: * corresponds to a significant difference (*p* < 0.05) between Stable and Increase groups

#### Negative life events

Negative life events between T0 and T1 were measured with an expanded version (20 items) of the Brugha List of Threatening Experiences [28, 29]. Participants indicated the presence or absence of an event during the 12-month period between baseline T0 and T1. The sum of negative life events was calculated and used as a continuous measure in the analyses.

#### Experience sampling procedure

Time-series data on affect states and daily unpleasant events were collected by means of experience sampling methodology [13, 30]. Participants filled in short questionnaires on a PsyMate™, a custom-made electronic device (www.psymate.eu), for 6 days, 10 times a day at semi-random moments between 07:30 am and 10:30 pm. More details about the ESM procedure in the TWINSSCAN cohort can be found elsewhere [24].

### ESM measures

#### Positive and negative affect

We constructed negative and positive affect scores based on the mean item scores of all available assessed affect items. For the negative affect score, the mean score of all available negative affect items (“insecure,” “lonely,” “anxious,” “irritated,” “listless,” “suspicious,” “down,” and “guilty”) was used. For the positive affect score, the mean score of all available positive items (“cheerful,” “relaxed,” “satisfied,” and “enthusiastic”) was used. All items were formulated as follows: “At this moment I feel … (‘lonely’, etc.)” and assessed with 7-point Likert scales from 1 (“not at all”) to 7 (“very much”).

#### Daily unpleasant events

Daily events were recorded at every beep with a question about the most important event since the last beep and how pleasant/unpleasant this event was. Participants were asked to rate an event (if any) on a 7-point Likert scale ranging from − 3 as “very unpleasant” and 3 as “very pleasant”. For our study, we only used events that were appraised unpleasant or neutral (reference category).

#### Speed of affect recovery

We operationalized the speed of recovery as the amount of time it takes until the effect of unpleasant events on negative/positive affect is no longer significantly different from the person-specific mean of negative/positive affect.

### Analysis

#### Differences between groups in speed of affect recovery from daily unpleasant events

The speed of affect recovery was assessed by modeling the effect of unpleasant events on the level of negative and positive affect. These models were constructed for five time points, starting from the same time point, with the level of affect at time (*t*) as an outcome and unpleasantness of the event at the same time point (*t*) as a predictor. Following the contemporaneous association, the second model assessed the lag-1 effect (affect at time (*t*) as an outcome and the lagged unpleasantness of the event at the previous time point (*t* − 1) as predictor, approximately 90 min earlier), and so on, for five time points (*t*, *t* − 1, …, *t* − 4) in total. The reason to choose only five time points was the reduction of the number of observations due to restriction of the assessments within the same day (associations from 1 day to the next were omitted because of the large gap during the night).

Our ESM data had a multilevel structure: multiple observations (level 1) belonged to one person (level 2), and multiple people sometimes belonged to the same twin pair (level 3). Therefore, we used linear mixed models that are multilevel models including both fixed and random effects.

The general model equation (including only fixed effects) is presented below: (1):

1 $$ Level\ of\ affect= level\ of\ unpleasantness\ of\ the\ even{t}^{- lag}+ gender+ age+ time; $$

Before model estimation, negative and positive affect scores were person-mean centered by calculating the mean score for each individual and subtracting this score from the affect score at every time point. This was done in order to keep only within-person and not between-person changes in the models. As mixed error-component models were used, the following random effects were specified: on the individual level, the random intercept was added to correct for the different mean levels of the affect for the participants, and a random slopes for time and the event unpleasantness variables, to correct for possible individual linear trends in these variables over time. On the twin level, a random intercept was modeled to correct for possible differences in the effect due to belonging to the same twin pair. For the random effects, a diagonal positive definite matrix structure was used (meaning that random effects are not correlated with each other), and for the residuals, autocorrelation structure of order 1 (continuous AR (1)) with a continuous time covariate was used (meaning that we expect residuals to be correlated with themselves at previous time points). Both covariance matrix structures were chosen based on the model comparisons, as they were associated with the best model fit based on the Akaike information criterion (AIC). All models were corrected for age and gender. All analyses were conducted in R version 3.6.1 with the “nlme” package [31] (see Additional file 2 for R script). In addition, we checked whether mean levels of the used variables did not significantly differ between the two groups, to ensure valid comparisons in speed of recovery. To test the influence of different group compositions based on different cutoffs for the SCL-90 change score, we performed a limited version of multiverse analysis (based on [32]). For details, see Additional files 1 and 2.

As we aimed to examine the difference between groups in the speed of recovery, we investigated whether this effect differed between the Increase and Stable group at every time point. For that, we added an interaction effect of group*event to Eq. (1) that lead to Eq. (2) and fitted these models to the whole sample.

2 $$ Level\ of\ affect= level\ of\ unpleasantness\ of\ the\ even{t}^{- lag}+ level\ of\ unpleasantness\ of\ the\ even{t}^{- lag}\ast group+ group+ gender+ age+ time; $$

After that, we assessed the effect for each group separately, to assess group-specific trajectory of affect recovery. For that, models (Eq. (1)) were fitted separately for the Stable and the Increase groups for 5 consecutive time points.

#### Speed of affect recovery from daily unpleasant events as predictor of individual symptom trajectories

After estimating the group differences in speed of affect recovery, we investigated whether these estimates of the speed of recovery can predict future individual symptom trajectories. To create this personal indicator, we first fitted the multilevel models (I) for the whole sample, and then derived the random slope estimation of the variable “event unpleasantness” for each individual. Since the random slope represents the individual deviation from the mean regression slope, these estimations may be used as a proxy for the effect for each individual. We extracted these random slopes for the model at contemporaneous (*t*) time point, *t* − 1, and so on, based on the results of the previous (group-based) analysis (see the “Results” section). Thus, we had several scores for each individual, representing the individual effect of the event unpleasantness on affect at *t*, *t* − 1, and so on. After that, we combined these several scores into one affect recovery measure. To do so, we used these individual scores to calculate individual areas under the curve with respect to baseline (AUCb) using the formula proposed by Pruessner and colleagues [33]. Thus, steeper recovery curve would mean smaller AUCb and faster affect recovery, and less steep recovery curve would mean larger AUCb and slower affect recovery. After that, we tested whether these individual AUCbs were associated with the SCL-90 scores at T1, corrected for scores at T0, belonging to twin pair (as a random intercept), age, gender, and number of negative life events from T0 to T1. For the effect size estimation, the outcome and predictor variables were standardized using a grand mean score (see Additional file 2 for R script).

## Results

### Sample characteristics

**Fig. 1:**
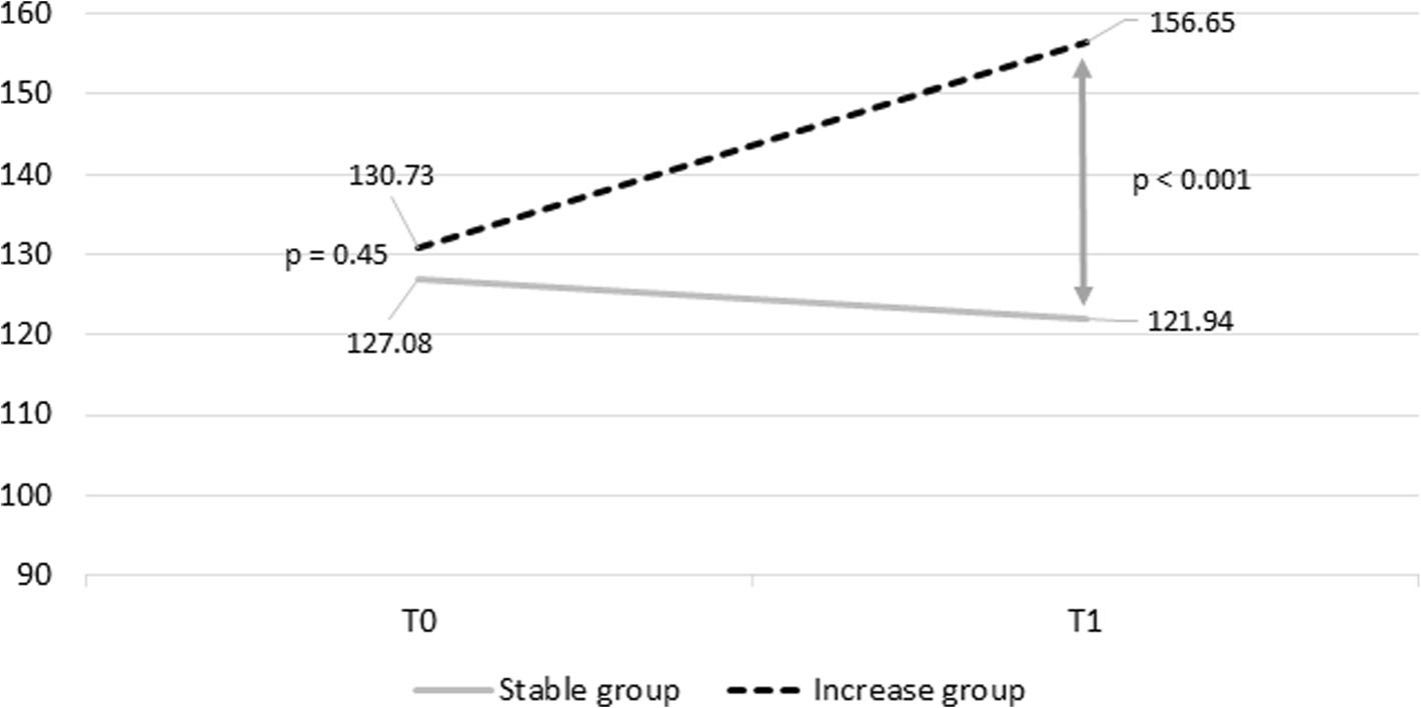
The change in SCL-90 mean sum score for the Stable and the Increase groups. In this figure, the *y*-axis represents the total sum score of the SCL-90 items; *x*-axis represents the baseline (T0) and 1 year (T1) assessments. The lines represent the change in the number of symptoms for the Stable group (solid light-gray line) and for the Increase group (dashed dark-gray line). *p* values correspond to the results of the tests of the differences of SCL-90 sum-scores between the Stable (*n* = 80) and the Increase (*n* = 77) groups at T0 and T1. The Stable and the Increase group did not differ significantly on the SCL-90 score (difference = 3.65, *p* = .45) at T0. At T1, the level of symptoms of the Increase group was significantly higher than of the Stable group (difference = 34.72, *p* < 0.001) which roughly corresponds to an increase of one severity category [34]

In line with our previous paper on the same subsample [24], 839 individuals had enrolled in T0. From them, 25 people (2.98%) had no JTV data and were excluded. Then, we selected the subsample with a lower level of happy childhood experiences based on the median split of JTV scores resulting in a sample of 451 individuals. Among the remaining 451 individuals, SCL-90 data on both T0 and T1 were available for 249 participants (4 participants missed the SCL-90 data at baseline, and 200—at follow-up, 44.25% drop-out). From the remaining subsample, ten participants were excluded because they provided less than 30% of ESM data (4.01%), and two because they reported no negatively appraised daily events (0.84%). This resulted in 237 participants. When grouped based on tertiles of change in the SCL-90 sum score in 1 year follow-up, this led to three groups: one (Stable group) of 80 participants that showed the smallest change in symptoms (for details see Table 1); one (Increase group) of 77 participants that showed the largest increase in symptoms (for details see Table 1), and one (Decrease) group of 80 participants (*M*_age_ = 17.84, age range 14–33 years, SD = 3.84; 66.25% females) who showed the largest decrease in symptoms. As the latter subgroup had significantly higher SCL-90 scores at baseline than the other two groups (*p* < .0001 with the comparison to the Stable group and *p* < .0001 with the Increase group), this group was excluded from analyses. The Stable and the Increasing group did not differ significantly on the SCL-90 score (difference = 3.65, *p* = .45) at baseline. At T1, the level of symptoms of the Increase group was significantly higher than of the Stable group (difference = 34.71, *p* < 0.001) which roughly corresponds to an increase of one severity category [34]. Trajectories of psychopathology for the two groups are presented in Fig. 1.

The Stable and Increase groups did not significantly differ in sociodemographic characteristics and mean level affect and number and level of unpleasant events (Table 1). Groups also did not differ in the number of occasions when unpleasant events occurred at two and three consecutive time points.

The Increase group had significantly lower level of happy childhood experiences (JTV) (difference = 0.45, *p* = 0.04).

In Table 1, JTV is 4 items (“I had a happy childhood,” “my parents greatly loved each other,” “I got the attention that I needed,” and “my privacy was respected”) from Dutch questionnaire on adverse childhood experiences [25]. SCL-90 is from Symptom Check List-90 (SCL-90) questionnaire [27], sum score of all items. Number of negative life events between T0 and T1 is measured with Brugha List of Threatening Experiences [28].

### Differences between groups in speed of affect recovery from daily unpleasant events

**Fig. 2:**
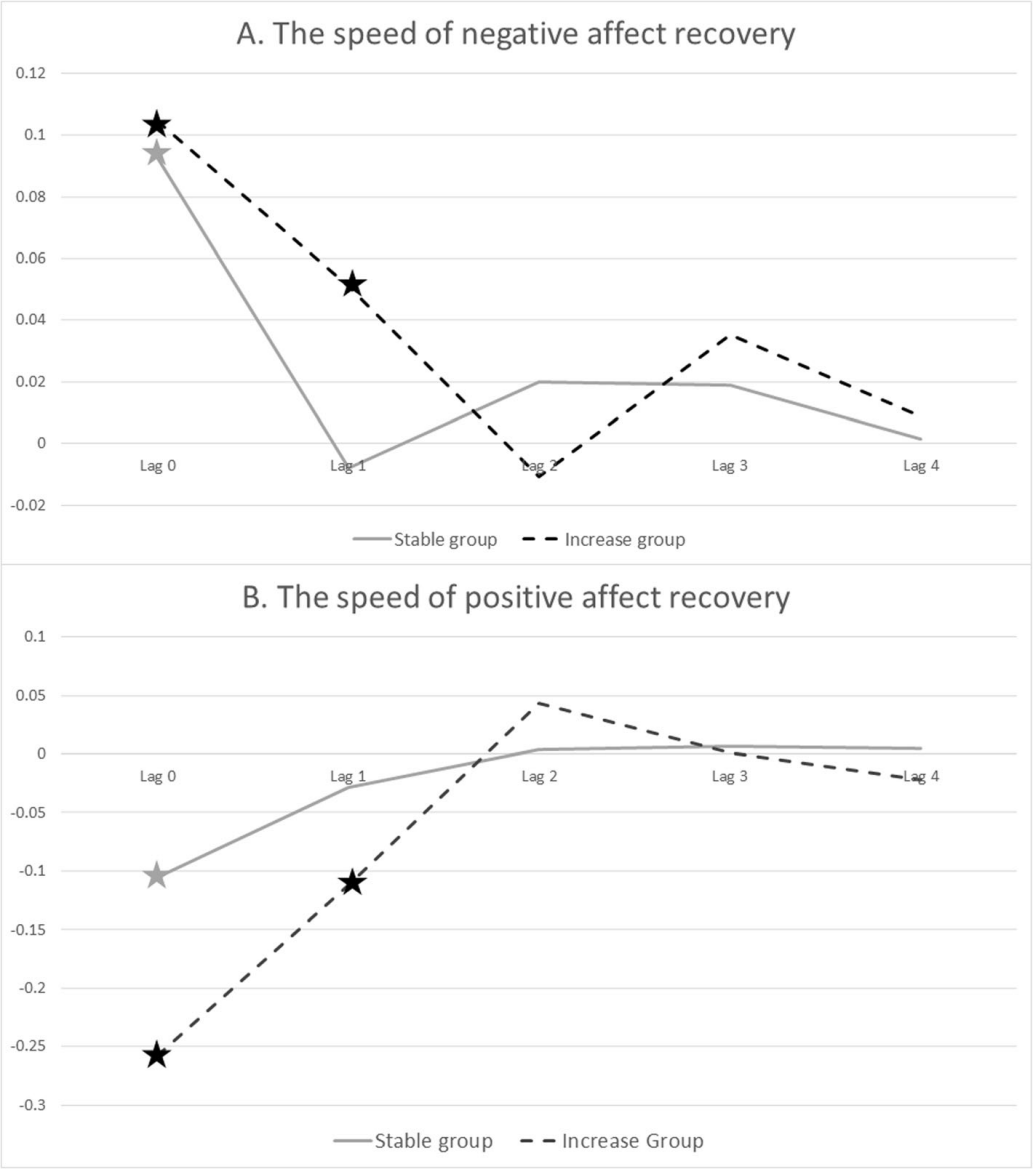
The speed of negative and positive affect recovery for Increase and Stable groups. In these figures, the *y*-axis depicts the b-coefficients that represent the effect of affect (negative for **a**, positive for **b**) from the model at the corresponding lag after the unpleasant events. Lag 0 corresponds to the contemporaneous association at the moment of the unpleasant event, and lags 1–4—the associations 90, 180, 270, and 360 min, respectively, between the event and affect. The solid gray line represents the pattern of recovery of negative affect for the Stable group, and the dashed black line represents pattern of recovery of negative affect for the Increase group. Stars indicate significant (*p* < 0.05) differences for the level of affect from person-specific mean levels of affect

At the same time point (lag 0), there was no significant difference between the groups in the effect of unpleasant events on negative and positive affect. For both groups, the effect was present (see Table 2 and Fig. 2).

**Table 2:** The effect of unpleasant events on negative and positive affect, per group and group * unpleasant event interaction

	The Stable group				The Increase group				Interaction effect			
	Negative affect		Positive affect		Negative affect		Positive affect		Negative affect		Positive affect	
	*B*	*p*	*B*	*p*	*B*	*P*	*B*	*p*	*B*	*p*	*B*	*p*
Lag 0	0.09*	< 0.01	−0.11*	< 0.01	0.11*	< 0.01	−0.15*	< 0.01	0.02	0.34	−0.05	0.19
Lag 1	−0.01	0.62	−0.03	0.30	0.05*	< 0.01	−0.08*	< 0.01	0.05*	0.02	−0.06	0.15
Lag 2	0.02	0.25	0.00	0.87	−0.01	0.62	0.04	0.19	−0.03	0.33	0.03	0.39
Lag 3	0.02	0.38	0.01	0.83	0.04	0.17	−0.01	0.85	0.02	0.59	−0.02	0.72
Lag 4	0.00	0.95	0.00	0.88	0.01	0.70	−0.03	0.44	0.02	0.59	−0.04	0.43

*indicates significant (< 0.05) effect

At the next time point (average 90 min after the event, lag 1), the groups differed with regard to the effect of unpleasant events on negative affect, but not positive affect (see Table 2 and Fig. 2). Stratified by group, for both negative and positive affect, the effect remained detectable for the Increase group, but not for the Stable group. To check robustness of the group difference with regard to the lag-1 effect of unpleasantness on negative affect, a limited multiverse analysis was performed (based on the idea by [32]), which suggested robustness of the effect to different group compositions. For details, see Additional file 1.

At the following time points (lags 2, 3, and 4), the effect was no longer significant for neither negative nor positive affect, and there were no differences between groups (see Table 2).

### Speed of affect recovery from daily unpleasant events as predictor of individual symptom trajectories

**Fig. 3:**
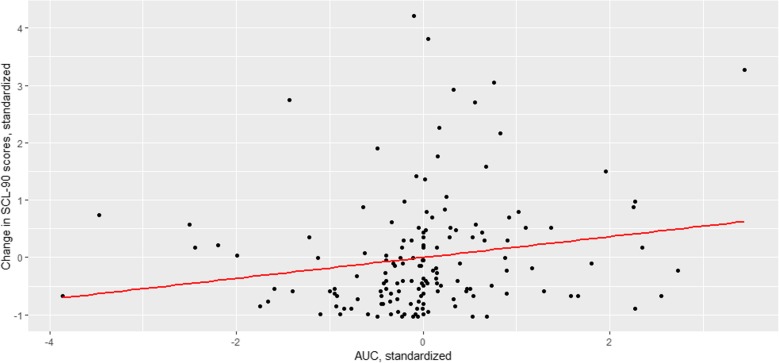
Speed of negative affect recovery as a predictor of individual symptom trajectories. In this figure, the *y*-axis depicts the standardized score of the change of SCL-90 from baseline to follow-up. 0 represents the mean change in Stable and Increase group (i.e., increase of 10.21 points), and each step of 1 corresponds to 1 SD increase (or decrease) in the SCL-90. *x*-axis depicts the standardized score of area under the curve with respect to baseline (AUCb) of the negative affect recovery after the negatively appraised events: 0 represents the mean AUC, and the step of 1 SD increase (or decrease) in the AUCb. Thus, smaller values indicate smaller AUCb and therefore *faster affect recovery*, and larger values represent larger AUCb and therefore *slower affect recovery.* The fitted line represents the linear relationship between change in SCL-90 scores and speed of affect recovery, depicting a small positive correlation between these variables, meaning that the increase in the level of SCL-90 is positively correlated with the longer affect recovery

As the difference between groups was detectable on *t* – 1, we extracted the random slopes for the model at contemporaneous (*t*) time point, *t* − 1 and *t* − 2 (one more to represent the recovery to baseline). For negative affect, resulting AUCb was borderline significantly (Beta = 0.09, *p* = 0.051) associated with SCL-90 scores on T1 (corrected for scores at T0). For positive affect, AUCb was not associated with SCL-90 scores on T1 (Beta = − 0.02, *p* = 0.74) (Fig. 3).

## Discussion

This study examined whether the speed of affect recovery from small perturbations in daily life differs between adolescents with different mental health trajectories in the following year. Results show that, in individuals who will develop more symptoms in the following year, their negative affect recovered slower after unpleasant events than in people with no increase in symptoms in the following year. For positive affect, group differences were not significant. Stratified by group, the effect of unpleasant events on both negative and positive affect was detectable 90 min longer in the Increase group than in the Stable group. The analysis of the individualized estimations of speed of recovery similarly showed an association between speed of negative affect recovery and individual future symptoms change, but did not reach significance.

### Complex systems principles and psychopathology

This study supports the idea that the complex systems approach can be applied to psychopathology. This approach assumes that the system can shift between alternative states, such as between having different levels of symptoms. These results add to the growing body of research suggesting that dynamic indicators of stability of complex systems, which estimate the process of “critical slowing down,” may be also applicable to mental health. The current results have shown that a direct measure hereof—namely speed of affect recovery from small perturbations—predicted mental health outcomes. Moreover, although at baseline the two groups were similar in levels of symptomatology, they already differed in this dynamic measure of resilience. Thus, the dynamic examination of speed of recovery may capture some additional information compared to simple mean levels of stressors, affect states, and levels of symptoms. Therefore, in the future, a complex systems approach to mental health may contribute to a more accurate and reliable prediction of risk and resilience in psychopathology.

### The dynamic concept of resilience

Psychological resilience is a popular topic in contemporary mental health research, as many scholars believe that focusing on protective mechanisms may yield insights for prevention and treatment [35–37]. However, most studies attempt to examine resilience using static measurements, such as retrospective questionnaires estimating personal competences, acceptance of change, social abilities and support, coping strategies, levels of optimism, and meaning in life [38–40]. However, the concept of resilience, in most of its definitions, is about people’s ability to withstand adverse circumstances, making the concept a dynamic one [11, 41, 42]. Although static measures certainly may tap into important aspects of resilience, they are unlikely fit to fully capture a dynamic concept. Defining resilience from a complex systems perspective has the advantage that it can be assessed in a direct, dynamic way, by prospectively measuring the impact of minor perturbations on the system. Although replication is warranted, the dynamic assessment of resilience may become a valuable tool to assess and monitor change in psychological resilience both for research and clinical practice.

### Methodological issues

The current study has several methodological issues. First, as the data came from a twin sample, it is possible that twins may have different dynamics of affect than non-twins, and therefore the findings may not be fully generalizable. Moreover, the phenotype of slower (or faster) affect recovery may have a shared hereditary component. However, despite being a twin cohort, we could only use those participants who also had follow-up measurements. Thereby, although interesting, this sample is strongly underpowered for any hereditary investigations. Second, the approach that we took for creating individualized affect recovery indicators has both benefits and limitations. The additional benefits of this approach were (i) the creation of one indicator that reflected recovery over several time points, (ii) a possibility to test the predictive value of this indicator on the individual level, and (iii) a possibility to obtain potentially clinically relevant estimations of effect sizes (i.e., how differences in the speed of recovery were associated with change in SCL-90 scores). The limitation of this approach, however, was a reduction of power due to the loss of the multilevel structure of the data, as this approach was performed with one score representing the speed of recovery per individual (although the time-series data allowed us to retain more power due to the lower standard deviations of the variables which were constructed based on multiple observations, compared to a hypothetical cross-sectional study with only one variable per person). Therefore, the borderline significance of the association between this AUCb score and future level of symptoms may be also due to the lack of power. Finally, symptom trajectories were measured with only two assessments, 1 year apart, which adds much noise to the data. Therefore, the results of this study should be considered preliminary until reproduced with more data observations and higher temporal precision.

### Clinical translation and future directions

The above method of assessing people’s current resilience state may have clinical value, not only as a way to monitor individual resilience but also as a new potential target for intervention and prevention strategies. There are, however, some important steps in the process of translating this study outcome to clinical practice. First, findings need to be translated from the group level to the individual level. The differences between individuals concerning affect dynamics may be substantial [43] and it is very important to investigate which changes are of clinical relevance and for whom. The results of this study represent the average effect over many, and therefore the overall effect is an average of individual differences in affect dynamics. Moreover, individuals may also differ in the moment when they precisely developed symptoms, and this moment was not assessed in the current study as only a single follow-up measure was used. Thus, new personalized designs, in which people are continuously and intensively monitored with regard to daily stress, affect and symptoms over extended periods of time, are required to establish whether CSD indicators indeed consistently anticipate relevant symptom changes. Although our study represents a first step towards testing this hypothesis, an important next step is to reproduce these findings at the individual level.

Second, we can assume that speed of recovery, as an indicator of system stability, is not a constant but will change over time. If we thus want to monitor changes in people’s resilience, we should measure how the speed of recovery from daily unpleasant events *changes over time* within individuals. This would require a design in which individuals are monitored with ESM over a longer period of time (e.g., several months). Feasibility of such designs in patients has recently been established (unpublished communication).

Finally, for this study, we assume that CSD, because it signals instability of the system, is relevant in predicting vulnerability to psychopathology. With the current design, it was not possible to assess directly whether a sudden transition occurred and, if so, at what moment in time. Therefore, for future studies, it is important to attempt to follow participants through transitions between states and to directly assess the timing and shape of this transition and the changes in the speed of recovery with respect to them.

## Conclusions

This paper applies complex dynamic systems theory to mental health and is the first to demonstrate that a direct indicator of critical slowing down—speed of recovery from small perturbations—may predict mental health problems in the following year, over and above the level of symptomatology. The paper supports the notion that mental health may behave according to the laws of a complex dynamic system and provides a basis for the use of a new dynamic measure of psychological resilience. This dynamic measure may have useful clinical applications.

## Additional files

Additional file 1 Description of limited multiverse analysis.

Additional file 2 R script, R.

## Abbreviations

AUCb: Area under the curve with respect to baseline
CSD: Critical slowing down
ESM: Experience sampling methodology
JTV: Dutch questionnaire on adverse childhood experiences (Jeugd Trauma Vragenlijst)
SCL-90: Symptom Check List-90
