# Supplementary material for: Measuring resilience prospectively as the speed of affect recovery in daily life: a complex systems perspective on mental health
Source: BMC Med. 2020 Feb 18;18:36. doi: 10.1186/s12916-020-1500-9 (PMC7027206; doi:10.1186/s12916-020-1500-9)
Supplement: Supplementary file 1 — Description of limited multiverse analysis. [file 12916_2020_1500_MOESM1_ESM.docx]

**Limited multiverse analysis**

In the current work, the group allocation was based on the tertiles of the change in SCL-90 symptoms. The reason behind such allocation was to use as many particiapnts as possible, and came up with the groups with same starting level of symptoms and different future symptoms trajectories. However, the cut-off between groups and precise group allocation was somewhat subjective and depended on the algorithm used in the R-function (*xtile* from ‘stata’ package by M. Gomez, [link](https://cran.r-project.org/web/packages/statar/statar.pdf)). Therefore, there were many alternative possible group allocations with different cut-off scores.

Thus, to explore to what extend the cut-off for the SCL-90 scores for the creation of the different groups influences the results, we performed a limited multiverse analysis (based on Steegen et al., 2016), restricted only to different choices with regards to group allocation. To do that, we needed to repeat the analysis for all possible group allocations. In order to simplify the process, we also restricted the analysis to one model for the time point at which the difference between groups was statistically significant. Thus, we tested all possible combinations of potential alternative “Stable” and “Increase” groups based on the different cutoff scores of SCL-90 change, with the following parameters: (i) the groups should have at least 70 people and (ii) have comparable levels of SCL-90 scores, affect and unpleasant event levels at baseline, and (iii) different levels of SCL-90 scores at follow-up. After that, the p-values obtained from these analyses were plotted and the distribution was assessed visually based on following principle: If an effect is absent, the p-values distribution is expected to be flat, because each p-value is equally probable in the absence of an effect. If an effect is present, distribution will be skewed to the zero, as probability of low p-values is higher.

Results: There were 29 possible group allocations. As the Stable and Increase groups differed significantly in the effect of unpleasant events on negative affect at t-1, we have modelled the group difference in the effect of unpleasant events on negative affect at t-1 (using the equation II, see the main document) for all 29 groups. This resulted in 29 p-values, 11 of them were <0.05. The distribution of p-values is presented in figure s1. The figure shows the skewed to zero distribution, which is interpreted as robustness of the effect to subjectivity of the group allocations

*Figure s1. Distribution of p-values for the group difference in the effect of unpleasant events on negative affect at t-1 for all 29 possible groups.*


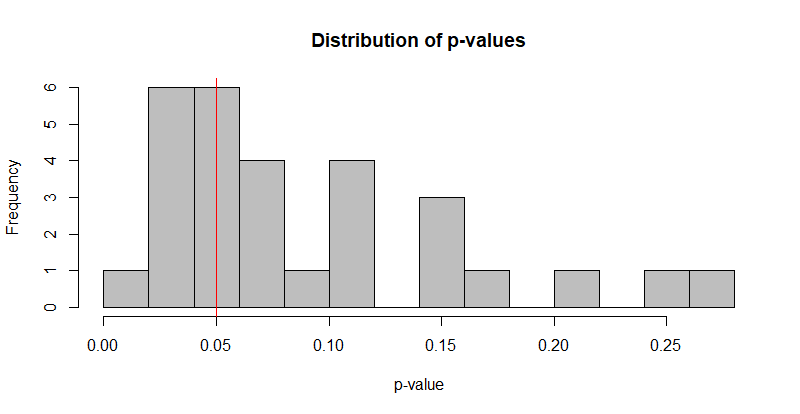


In this figure, the x-axis depicts the size of the p-values obtained from the model of the effect of unpleasant events on negative affect at t-1 for 29 possible groups, and the y-axis depicts frequency of these p-values.

References:

1. Steegen S, Tuerlinckx F, Gelman A, Vanpaemel W. Increasing Transparency Through a Multiverse Analysis. Perspect Psychol Sci. 2016.
